# The determination and analysis of the complete mitochondrial genome of *Dario dario* (Anabantiformes: Badidae)

**DOI:** 10.1080/23802359.2021.1981164

**Published:** 2021-09-27

**Authors:** Qi Wang, Xinyu Zhao, Chuanchen Ren, Fang Meng, Hongqiang Wang, Yifan Liu, Bingjian Liu, Enshang Yang

**Affiliations:** a National Engineering Research Center for Marine Aquaculture, Zhejiang Ocean University, Zhoushan, China; b National Engineering Laboratory of Marine Germplasm Resources Exploration and Utilization, Marine Science and Technology College, Zhejiang Ocean University, Zhoushan, China; c Zhoushan Hospital of Zhejiang Province, Zhoushan, China; d School of Marine Engineering Equipment, Zhejiang Ocean University, Zhoushan, China

**Keywords:** *Dario dario*, Badidae, Anabantiformes, mitochondrial genome, phylogenetic relationship

## Abstract

The classification of Badidae family based on morphology has been revised several times, but data on complete mitogenome are scarce, the complete mitochondrial genome of the Badidae fish *Dario dario* was characterized for the first time in the present study. The whole mitogenome was 16,830 bp in size and consisted of 13 protein-coding genes, 22 tRNAs, two rRNAs genes, a control region and origin of light-strand replication. The proportion of coding sequences with a total length of 11,431 bp was 67.92%, which encoded 3800 amino acids. The genome composition was highly A + T biased (58.12%), and exhibited a negative AT-skew (–0.0045) and GC-skew (–0.2347). All protein-coding genes started with ATG except for GTG in CO1, while stopped with the standard TAN codons or a single T. The control region (D-loop) ranging from 15,658 bp to 16,830 bp was 1173 bp in size. Phylogenetic analysis showed that *D. dario* was most closely related to *Badis badis*. The complete mitochondrial genome sequence provided new insight into taxonomic classification, and a more complex picture of species diversity within the Anabantiformes.

Badidae is known as a small family (about 30 species), a few species have been described since, and the family currently comprises six species of *Dario* (Rüber et al. 2004) and about 20 species of *Badis* (Basumatary et al. 2016). *Dario dario* (Hamilton, 1822), has been positioned as Badidae. It is a small predatory fish, mainly distributed in southeast of Asia and India. Specimens of *D. dario* collected by Zhejiang Engineering Research Center for Mariculture and Fishery Enhancement Museum (PH18146) from Yamuna River of New Delhi (28°36′50″N, 77°12′30″E) were identified by both the morphological features and the COI. Tissue samples stored at −20 °C were preserved in 95% ethanol and total genomic DNA was extracted from muscle using the phenol–chloroform method (Barnett and Larson 2012). Sequences were amplified by PCR with long and accuracy Taq (LA-Taq) DNA polymerase (Takara, Tokyo, Japan‎) following the manufacturer’s protocol and assembled by CodonCode Aligner 5.1.5 (CodonCode Corporation, Dedham, MA). The primers (Table S1) used in this study are the universal primers designed in conserved regions. Transfer RNA genes were generated by the program tRNAs-can-SE (Lowe and Eddy 1997); the composition and the relative synonymous codon usage (RSCU) were obtained using MEGA X (Kumar et al. 2018). The base compositional bias of the mitochondrial genome AT skew and GC skew was calculated using the formulae: AT-skew=(A − T)/(A + T); GC-skew=(G − C)/(G + C) (Perna and Kocher 1995).

Similar to the typical mitogenome of vertebrates, the mitogenome of *D. dario* deposited in GenBank (MT344964.1) is a closed double-stranded circular molecule of 16,830 bp including 13 protein-coding genes, two ribosomal RNA genes, 22 tRNA genes, and two main noncoding regions (Boore 1999). The overall contents of A, C, G, and T were 28.93%, 25.83%, 16.02%, and 29.19%. A-T and G-C contents were 58.12% and 41.88%, thereby with a high AT bias. Both AT-skew and GC-skew of the mitogenome were negative (–0.0045, −0.2347). Most mitochondrial genes are encoded on H-strand except for ND6 and eight tRNA genes encoded on the other complementary strand. In addition, 55 base pairs in 11 intergenic spacers were found in the *D. dario* mitogenome, ranging from 1 to 34 bp in length. Simultaneously, nine overlapping sites (totally 28 bp) were observed in both PCGs and tRNA genes. Among them, the largest overlap is 10 nucleotides, between ATP6 and ATP8. The lengths of 12S rRNA and 16S rRNA were 950 bp and 1694 bp, while the length of control region was 1173 bp, ranging from 15,658 bp to 16,830 bp.

Thirteen PCGs were 11,431 bp (67.92%) and encoded 3800 amino acids. Moreover, the AT-skew (–0.0733) and GC-skew (–0.2918) for the PCGs in *D. dario* were negative. All the PCGs used the initiation codon ATG except for GTG in CO1. Besides, CO_2_, ND4, CytB ended by single T, ND3 ended by TAG, all the others ended by TAA. The base content of nucleotides differed in the sense strands of the PCGs (T, 31.05%; A, 26.80%; G, 14.92%; C, 27.22%). The overall A + T content in the sense strands of the PCGs (57.85%) showed the obvious bias in the AT nucleotide composition. The values of RSCU showed that Leu2; Val; Ser1; Pro; Thr; Ala; Arg; Gly were higher codon usage at the same level encoded by four synonymous codons, while the others were lower codon usage encoded by either three or two codons.

The lengths of 12S rRNA and 16S rRNA were 950 bp and 1694 bp, it showed a positive AT skew (0.2138) and negative GC skew (–0.0755). The total length of the 22 tRNAs in the *D. dario* mitochondrial genome was 1553 bp, and the overall A + T content of tRNAs was 56.60%. It had a positive AT skew (0.1081), but negative GC skew (–0.1246). The length of CR was 1173 bp, ranging from 15,658 bp to 16,830 bp, 420 nucleotides for A, 385 nucleotides for T, both of them accounting for 68.63% of the whole D-loop, the AT and GC skew values were 0.0435 and −0.1957.

Based on the Akaike information criterion (AIC), GTR + G+I + F was indicated as the best-fitting substitution model for the phylogenetic relationship analysis. In Figure 1, it is obvious that *D. dario* was most closely related to *B. badis*; these two species formed a monophyletic clade with high support value constituting a Badidae group. Besides, Anabantidae + Helostomatidae + Osphronemidae forms a monophyletic clade, and formed sister branches with Badidae + Pristolepididae + Channidae. Phylogenetic analysis was used to get a clear understanding of classification status, and here better clarification of the phylogenetic classification of *D. dario.* The more discovery of these species will further promote more research on Badidaes.

**Figure 1.**
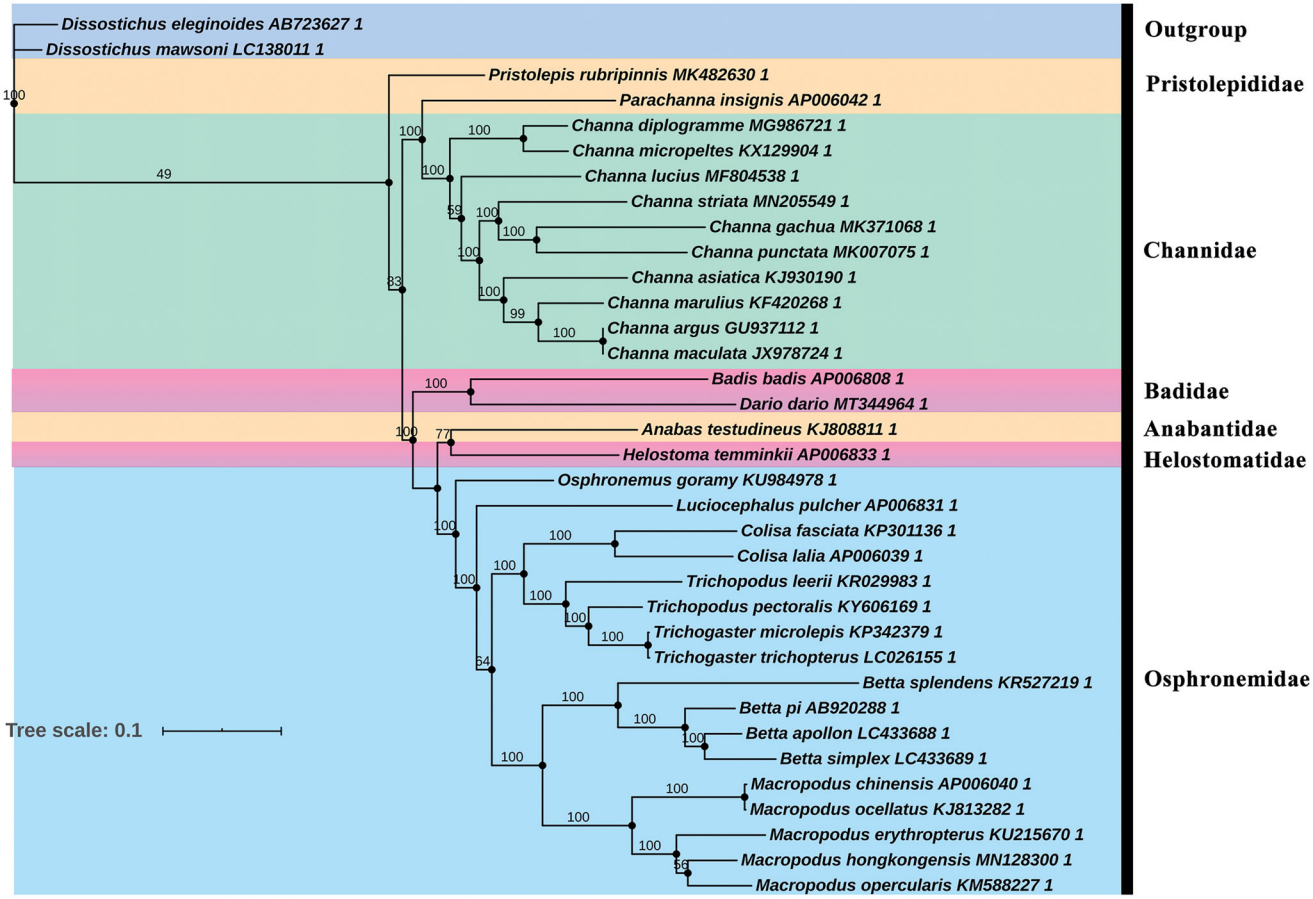
Phylogenetic tree of Anabantiformes species inferred from the 13 PCGs based on maximum-likelihood analysis. Support values for the maximum-likelihood analyses (bootstrap support with 1000 replications) are shown next to nodes. The number after the species name is the GenBank accession number.

## Supplementary Material

Supplemental Material

## Data Availability

The genome sequence data that support the findings of this study are openly available in GenBank of NCBI at https://www.ncbi.nlm.nih.gov/ under the accession no. MT344964.1.

## References

[CIT0001] Barnett R, Larson G. 2012. A phenol–chloroform protocol for extracting DNA from ancient samples. Methods Mol Biol. 840:13–19. 22237516 10.1007/978-1-61779-516-9_2

[CIT0002] Basumatary S, Choudhury H, Baishya RA, Sarma D, Vishwanath W. 2016. *Badis pancharatnaensis*, a new percoid fish species from Brahmaputra River drainage, Assam, India (Teleostei: Badidae). Vertebr Zool. 66:151–156.

[CIT0003] Boore JL. 1999. Animal mitochondrial genomes. Nucleic Acids Res. 27(8):1767–1780. 10101183 10.1093/nar/27.8.1767 PMC148383

[CIT0004] Kumar S, Stecher G, Li M, Knyaz C, Tamura K. 2018. Mega X: molecular evolutionary genetics analysis across computing platforms. Mol Biol Evol. 35(6):1547–1549. 29722887 10.1093/molbev/msy096 PMC5967553

[CIT0005] Lowe T, Eddy S. 1997. tRNAscan-SE: a program for improved detection of transfer RNA genes in genomic sequence. Nucleic Acids Res. 25(5):955–964. 9023104 10.1093/nar/25.5.955 PMC146525

[CIT0006] Perna NT, Kocher TD. 1995. Patterns of nucleotide composition at fourfold degenerate sites of animal mitochondrial genomes. J Mol Evol. 41(3):353–358. 7563121 10.1007/BF00186547

[CIT0007] Rüber L, Britz R, Kullander SO, Zardoya R. 2004. Evolutionary and biogeographic patterns of the Badidae (Teleostei: Perciformes) inferred from mitochondrial and nuclear DNA sequence data. Mol Phylogenet Evol. 32(3):1010–1022. 15354300 10.1016/j.ympev.2004.04.020

