# Supplementary material for: The determination and analysis of the complete mitochondrial genome of Dario dario (Anabantiformes: Badidae)
Source: Mitochondrial DNA B Resour. 2021 Sep 27;6(10):3073–5. doi: 10.1080/23802359.2021.1981164 (PMC8477940; doi:10.1080/23802359.2021.1981164)
Supplement: Supplemental Material [file TMDN_A_1981164_SM9951.docx]

| **Forward** | **Sequences (5′ to 3′)** | **Reverse** | **Sequences (5′ to 3′)** |
| --- | --- | --- | --- |
| **Dd-F0** | CTTAATTAAAGCATAACTCTG | **Dd-R0** | TTTCAGCGTTCCCTTGCGGTAC |
| **Dd-F1** | ACATGGTAAGTGTACCGGAAGG | **Dd-R1** | CTGGATTACTCCGGTCTGA |
| **Dd-F2** | AAGACGAGAAGACCCTATGGAG | **Dd-R2** | GAADGGBCCYCCNGCRTAYTCTAC |
| **Dd-F3** | GAACTAGTCTCAGGTTTCAA | **Dd-R3** | GGCGCTTAGCTGTTAACTAAG |
| **Dd-F4** | AGACCRAGGGCCTTCAAAG | **Dd-R4** | TARCTTCARTATCATTGRTGKCC |
| **Dd-F5** | TGGTACTGAAGTTACGAGTAC | **Dd-R5** | GGTTGATTTCGTAGGCCAA |
| **Dd-F6** | ACACCTTTACCCCAACCACGCA | **Dd-R6** | CCATATAAAGGTATCTTTACTA |
| **Dd-F7** | CTACAATGCTAAAARTYCTHATCCC | **Dd-R7** | TAGCCYHTGCTTGGATTTGCACCAAGAGT |
| **Dd-F8** | TCCAAGTAGTAGCTATGCACCC | **Dd-R8** | GGTTTGTATTGTTTACTTGTCAG |
| **Dd-F9** | ATCAARCGACTTGCCTGAGGAAG | **Dd-R9** | ATAGTTTAANTTBAGAATNCTAGCTTTGGG |
| **Dd-F10** | TTATTATTGCTGCCGTAAC | **Dd-R10** | GATANTAARGYYAGGACCAAACCTTTGTGC |
| **Dd-F11** | CACTCTTATTGAAGGTGAGGG | **Dd-R11** | CTTAACATCTTCAGAGTTATGCT |
| **Dd-F12** | ACATGGTAAGTGTACCGGAAGG | **Dd-R12** | TTTCAGCGTTCCCTTGCGGTAC |

**Table S1. Primers used for amplification and sequencing of the complete mitochondrial genome of *D. dario***
